# Identification of potential new COVID-19 treatments via RWD-driven drug repurposing

**DOI:** 10.1038/s41598-023-40033-8

**Published:** 2023-09-04

**Authors:** Yun Liao

**Affiliations:** Life Sciences, OptumInsight/OptumRx, UnitedHealth Group Inc, Basking Ridge, NJ USA

**Keywords:** Virtual screening, Drug development

## Abstract

By utilizing Optum Life Sciences Claims Data, we constructed Real World Data (RWD) cohorts comprising over 3 million patients and simulated a clinical trial observational study design to evaluate over 200 FDA-approved drugs with COVID-19 repurposing potential, and identified a dozen candidates exhibiting significant reduction in the odds of severe COVID-19 outcomes such as death, intensive care unit (ICU) admission, hospitalization and pneumonia. Notably, certain drug combinations demonstrated effects comparable to those of COVID-19 vaccines. Furthermore, our study revealed a novel finding: a quantitative linear relationship between COVID-19 outcomes and overall patient health risks. This discovery enabled a more precise estimation of drug efficacy using the risk adjustment. The top performing drugs identified include emtricitabine, tenofovir, folic acid, progesterone, estradiol, epinephrine, disulfiram, nitazoxanide and some drug combinations including aspirin-celecoxib.

## Introduction

The COVID-19 may have claimed greater than 18 million lives worldwide in just two years into the pandemic^1^, three times more than the reported global death toll, with more than 767 million confirmed cases worldwide as of May 2023^2^. Currently, about 30% of the world population, including 70% of people in low-income countries, remain unvaccinated against COVID-19^3^. With continuing worldwide-spread of the COVID-19 virus, the virus continues to mutate, and previous generations of COVID-19 vaccines are less effective against new variants. At present, there are over 9000 clinical trials^4^ worldwide aimed to develop new COVID-19 treatments but only a handful of COVID-19 therapeutics have been authorized under an emergency use authorization (EUA) or approved^5^. Among the approved antivirals (Paxlovid (nirmatrelvir/ritonavir), Lagevrio (molnupiravir), Azvudine (FNC), and Veklury (remdesivir)), Paxlovid stands out as a leading option due to its impressive 86% reduction in the risk of hospitalization or death when administered within three days of symptom onset^5^, but still faces challenges in terms of global accessibility, especially in developing countries, due to regulatory hurdles, economic limitations, and other barriers. Additionally, it is worth noting that Paxlovid has known interactions with certain commonly used medications and carries the risk of viral rebound after treatment^5^. SARS-COV-2-targeting monoclonal antibodies (Actemra (tocilizumab), REGEN-COV (casirivimab-imdevimab), Sotrovimab, Bamlanivimab-Etesevimab, Bebtelovimab, Evusheld (tixagevimab-cilgavimab)) under EUA, are no longer authorized to use in the US due to high frequency of variants^5^. Several immune modulators under EUA (Kineret (anakinra), Olumiant (baricitinib), Actemra (tocilizumab), Gohibic (vilobelimab)) can help suppress hyper-inflammation caused by COVID-19 for hospitalized patients but make patients more susceptible to infections due to immune-suppression^5^. Therefore, it remains highly desirable to find new COVID-19 treatments that are not only more efficient and safer but also more accessible and affordable to help combat and ultimately end the pandemic.

COVID-19 is a multi-systemic disease which affects almost all body organs, and the difference between people with asymptomatic or mild COVID symptoms vs severe COVID outcomes is not only due to the virus but also the host^6^. Therefore, it is important to investigate broad range of marketed medicines that target from virus life cycles to host factors or both for repurposing.

Testing new treatments via clinical trials is expensive and often takes many years to complete with high attrition rate due to failures. One of the biggest barriers to complete clinical trials is the shortage of participants. RWD-driven drug repurposing is a viable alternative, as this approach can quickly investigate tens or hundreds of drugs across millions of patients using vast existing clinical data.

Since the onset of the pandemic, scientists and physicians have worked together to find effective treatments against COVID-19, with a particular focus on repurposing drugs using computational and/or experimental approaches^7^, including molecular modeling, virtual screening, machine learning and AI, genetic associations, pathway network analysis, retrospective clinical analysis, high-throughput screening and clinical trials, which generated, altogether, over 200 repurposing drug candidates urgently waiting for real-world evidence (RWE).

Thus, we built over 200 drug study cohorts using data from > 3 million US patients exposed to COVID-19 prior to diagnosis with COVID-19 infection between 2020-01-01 and 2021-09-30 from the Optum Life Sciences Claims Dataset^8^. Next, we designed and conducted a series of RWD-driven analyses simulating clinical trial observational studies to investigate all potential repurposing drug candidates known to date (including candidates found from an in-house computational virtual screening molecular docking study of 1615 FDA approved drugs on SARS-CoV-2 3CL protease and RNA polymerase) for their real life impacts on five major COVID-19 consequences: infection, hospitalization, ICU admission, pneumonia and death. To counteract potential multiplicity errors caused by the multiple comparisons (multiple clinical trial end points), a Bonferroni Correction^9^ was applied when determining the statistical significance of drug efficacy by increasing confidence interval (CI) from 95 to 99%.

**Figure 1:**
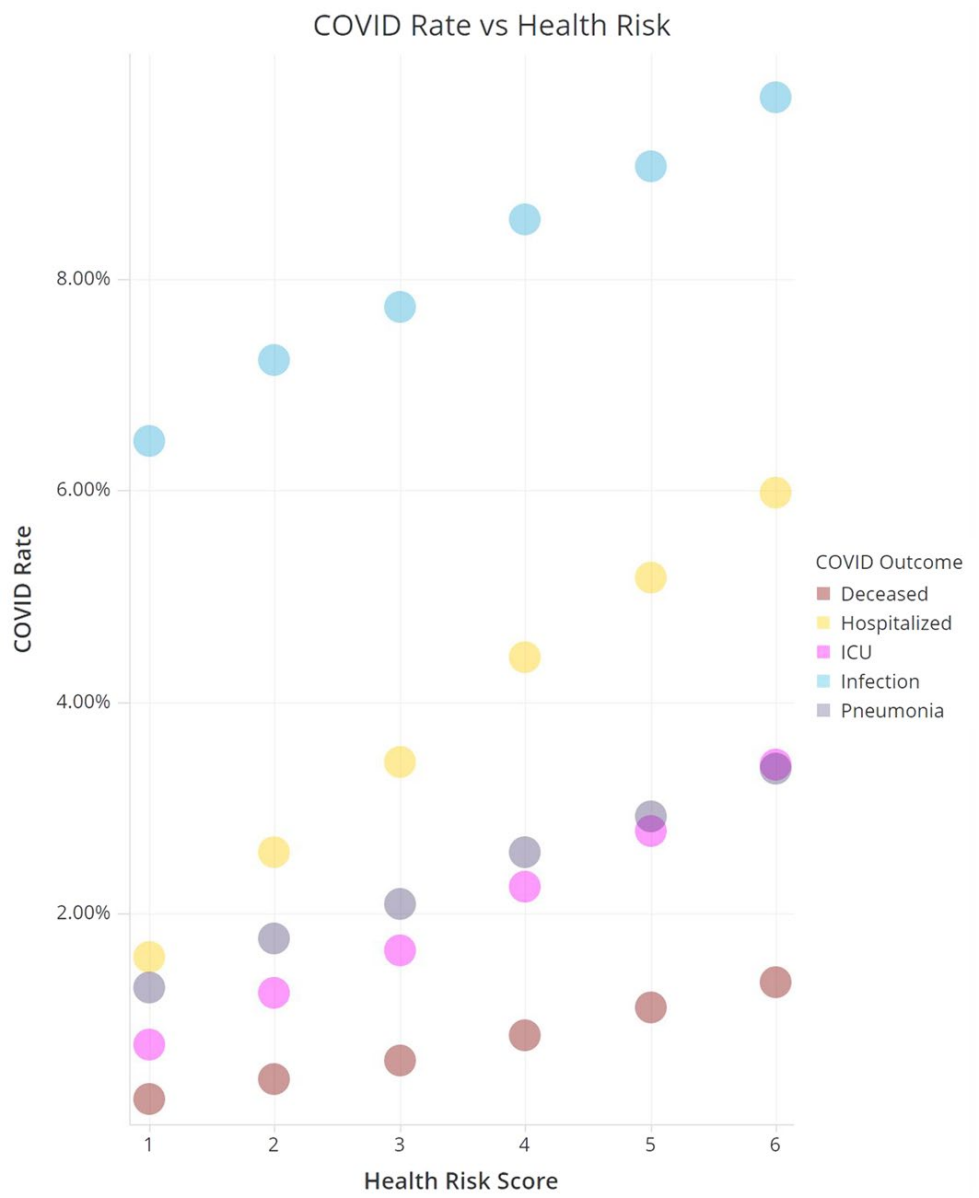
COVID-19 outcome rates vs ERG risk scores. Patients with risk score between n − 1 and n are categorized and grouped into risk score of n.

Significantly, a dozen FDA-approved small molecule oral drugs were identified with an odds reduction rate (ORR) (*ORR* = *(1 − odds ratio) *×* 100%, percentage drop of the COVID-19 outcome in the drug group vs the control group*) comparable to COVID-19 vaccines in reducing some severe COVID-19 outcomes. The result is summarized in Table 1. Briefly, 2,935,415 non-vaccinated patients were selected as a control group and 189,692 vaccinated patients were selected as a reference group. All drug groups include only non-vaccinated patients to avoid vaccine-drug interference. Instead of using common multivariate matching techniques like propensity score matching to reduce confounding and bias, we applied a unique risk adjustment approach using Optum Symmetry ERG^®^ Scores^10^ to make all drug groups and the control group comparable. The proprietary ERG Risk Scores were found highly related to COVID-19 outcomes in a simple linear regression pattern (Fig. 1 and Figs. S1–S5 of Supplementary section), thus enabling more straightforward and accurate drug efficacy estimation. As a result, several drugs stood out from drug classes including antivirals, hormones, vitamins and anti-inflammatories. The top repurposing drugs were summarized in Table 1 and more details including drug class, indication and repurposing rationales, p values, ERG risk scores and patient raw counts by COVID-19 outcomes, were summarized in Table S1 of Supplementary section.

**Table 1:**
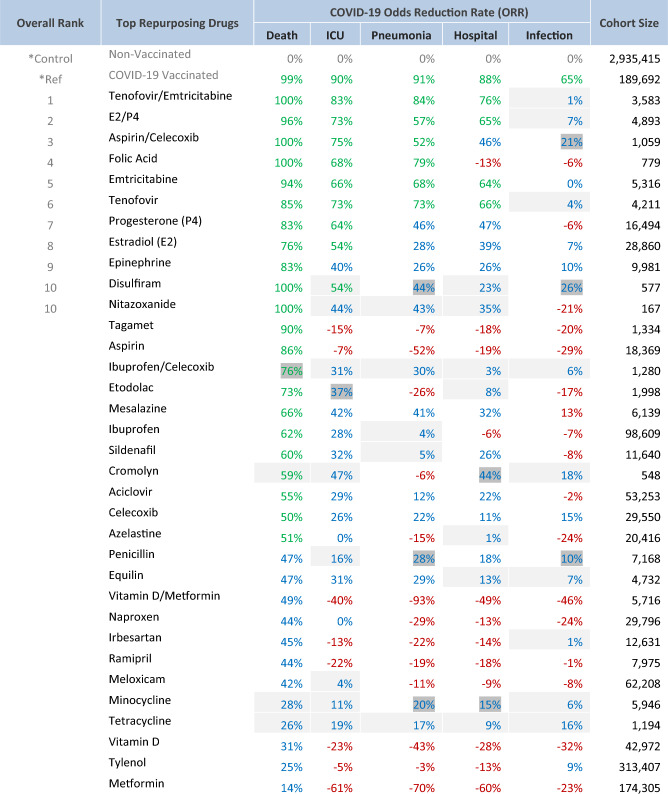
Top repurposing drugs—COVID-19 outcome odds reduction rate (ORR) vs control.

Green: ≥  50%; blue: < 50%; red: < 0%; drug combo: drug1/drug2; shallow grey background: statistically insignificant (95% CI one-sided); dark short grey background: statistically significant (95% CI one-sided) and insignificant (99% CI one-sided)). Statistical significance was calculated only for positive outcomes, of study interest and focus.

**Chart 1:**
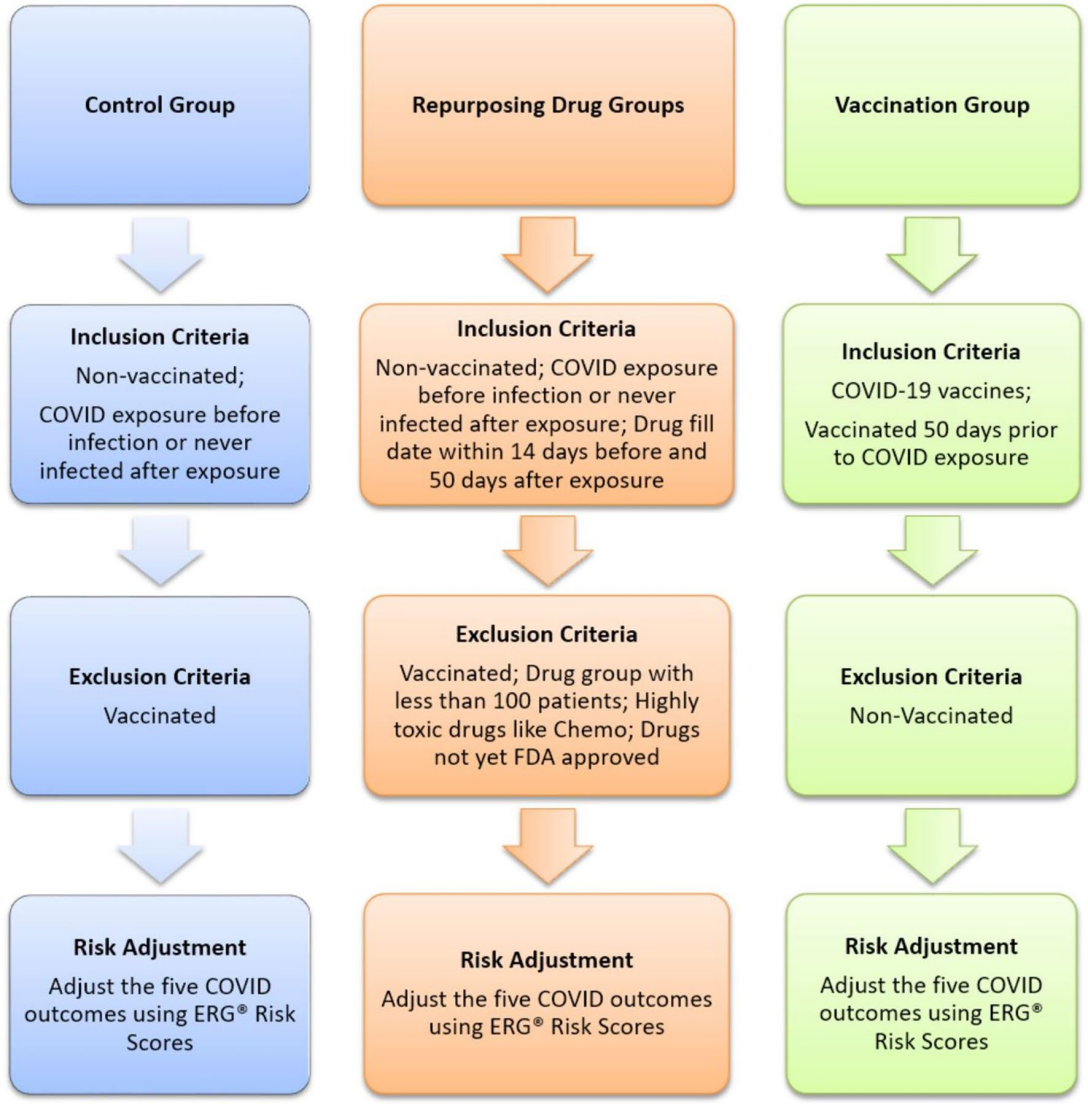
Study design flow chart.

Two anti-viral drugs, emtricitabine (5,316 patients) and tenofovir (4,211 patients), exhibited ORR (odds reduction rate) of 94% and 85% respectively on COVID-19-related deaths versus the control group (power 100.00%, p value < 0.0001), and their combination (3,583 patients) gained synergy to achieve 100% versus the control group (power 100.00%, p value < 0.00010) comparing to COVID-19 vaccines’ 99%. For other severe COVID-19 consequences like ICU admissions, pneumonia and hospitalization, the combination also achieved ORR of 83%, 84% and 76% respectively (power 100.00%, p value < 0.0001) comparing to COVID-19 vaccines’ 90%, 91% and 88% correspondingly. The drug repurposing rationale behind emtricitabine and tenofovir is that they may both inhibit SARS-CoV-2 RNA polymerase^11,12^.

Two sex hormones, progesterone (P4) (16,494 patients) and estradiol (E2) (28,860 patients), showed ORR of 83% and 76% respectively on COVID-19-related Death versus the control group (power 100.00%, p value < 0.0001) and their combination (4,893patients) gained synergy to achieve a 96% ORR versus the control group (power 100.00%, p value < 0.0001). For other severe COVID-19 consequences like ICU admission, pneumonia and hospitalization, the combination also achieved ORR of 73%, 57% and 65%, respectively (power 100.00%, p value < 0.0001). The drug repurposing rationale behind P4 and E2 is that they are both anti-inflammatory and immunomodulatory to mitigate the cytokine storm while increasing antibody production against COVID-19^13^. Also, estradiol may serve as a SARS-CoV-2 entry inhibitor^14^. E2 and P4 may have played an important role to the significantly lower risk of severe COVID-19 outcomes in women than men observed worldwide, and may provide an inexpensive, safe and easily accessible life-saving treatment for severe COVID^13^.

Another natural hormone, epinephrine (9,981 patients) also showed an ORR of 83% on COVID-19-related death versus the control group (power 100.00%, p value < 0.0001), which may be one of reasons why younger people are less susceptible to severe COVID-19 consequences, as they usually have much higher ratio of epinephrine vs norepinephrine than older people^15^. The drug repurposing rationale is based on epinephrine’s potential to regulate immune cell activity against COVID-19^16^.

Folic acid (vitamin B9) (779 patients), richest in peanuts by nature source, achieved an ORR of 100% (power 100.00%, p value < 0.0001) on COVID-19-related death, 79% (power 100.00%, p value < 0.0001) on pneumonia and 68% (power 99.97%, p value 0.0003) on ICU versus the control group. However, hospitalization ORR (−13%) is surprisingly negative. Notably, 95% of those hospitalized patients happened to have pregnancy-related complications during the study period, which may have been the primary cause of hospitalization rather than COVID-19 infection or folic acid which is commonly supplemented for pregnancy. The drug repurposing rationale comes from folic acid’s Immune-boosting capacity and potentials to inhibit SARS-CoV-2 RNA 3CL protease and spike protein cleavage^17^.

Several nonsteroidal anti-inflammatory drugs (NSAID) like aspirin, celecoxib and ibuprofen (Advil) all exhibited some degree of efficacy on some severe COVID-19 consequences, especially death. Significant synergy was observed in the aspirin and celecoxib combination (1,059 patients) achieving an ORR of 100% (power 100.00%, p value < 0.0001) on Death, 75% (power 100.00%, p value < 0.0001) on ICU and 52% (power 99.80%, p value 0.0017) on pneumonia versus the control group. The drug repurposing rationale is that aspirin deactivates platelets to reduce blood clots caused by COVID-19 and celecoxib may inhibit SARS-CoV-2 3CL protease, plus their anti-inflammatory effects against COVID-19^18^.

Some drugs that are already being used to treat COVID-19 patients in clinical practice did not make it onto our list of top repurposing drugs since they either did not perform significantly better than the control group, such as hydroxychloroquine, dexamethasone, heparin, ivermectin and etc., or did not have enough data available within the study window, such as remdesivir and nirmatrelvir/ritonavir.

In our study, the drug group patients usually have different level of existing health risks from those of the control group (Table S1, Supplementary Information), the higher the health risk, the higher risks/odds of more severe COVID-19 outcome observed usually, so a risk adjustment must be applied for more accurate efficacy calculation. We discovered that the COVID-19 consequences are often positively proportional to ERG® Retrospective Risks^10^ in a simple linear regression pattern across all study groups, which allows calculating odds reduction rates (ORR) more accurately using a more straightforward risk adjustment.

In summary, we have built simulated drug study cohorts using Optum claims data of over 3 million US patients and conducted a series of RWD-driven analyses simulating a clinical trials observational study design using Optum ERG® risk adjustment to quickly identify a list of FDA-approved small molecule oral drugs with promising RWE efficacy (odds reduction rate (ORR)) comparable or closer to COVID-19 vaccines in reducing severe COVID-19. This is also the first time that a quantitative linear relationship between COVID-19 outcomes and overall patient health risks was discovered. Our study using a unique design may be complementary to other COVID-19 drug repurposing efforts using RWD^19^, and results of the top drugs identified from this RWE study may support their further clinical trials validation in priority.

## Methods

### RWD drug repurposing design

Previous RWD studies have demonstrated observational studies can produce results similar to randomized controlled trials^20^. With this understanding we simulated a clinical trials observational study to explore potential drug repurposing for COVID-19 (Chart 1).

### Inclusion/exclusion criteria

Study window: 2020-01-01 ~ 2021-09-30.

### Exclusion

Drugs with serious side effects, such as chemotherapy drugs, or with less than 100 patients within the study window, or not FDA approved.

### Inclusion

#### Control group

2,935,415 non-vaccinated patients with documented COVID-19 exposure before infection diagnosed or never infected after exposure (ICD10 codes for COVID-19 Exposure: Z20.822; COVID-19 Infection: U07.1).

#### Vaccination group (gold standard)

189,692 patients vaccinated 50 days prior to documented COVID-19 exposure. The approved vaccination protocol requires 3 weeks between first vaccination and second. After the second inoculation, it usually takes another 4 weeks to achieve full protection^21^, 7 weeks in total (~ 50 days). Therefore, the 50-day criterion is to exclude patients exposed to COVID-19 before achieving full protection otherwise their COVID-19 consequences may not truly reflect the vaccine efficacy.

#### Repurposing drug groups

Non-vaccinated patients with COVID-19 exposure documented before infection diagnosed. Drug fill date within 14 days before and 50 days after COVID-19 exposure. The “14 days before” criterion allows instant drug protection effect against COVID-19 infection to be evaluated as COVID-19 may have an incubation period up to 14 days^22^. The “50 days after” criterion allows evaluation of the drug effect against all severe COVID-19 consequences developed up to 7 weeks after COVID-19 exposure.

All drug and control groups in this study exclusively consist of non-vaccinated patients due to the following reasons: (1) strong vaccination outcomes: Vaccination results are highly influential and robust, making it challenging to discern the effects of other drugs if they were combined within the same study. By focusing solely on non-vaccinated patients, we can specifically investigate the potential effects of different drugs without the confounding influence of vaccinations. (2) Differential medication schedules: Vaccines often require distinct administration schedules compared to medications used in the drug groups. For instance, vaccinations are typically effective when administered before infection, whereas other drugs may be effective both before and after infection due to different mechanisms of action. By limiting drug groups to non-vaccinated patients, we can obtain more precise insights into the specific effects of different drugs under investigation, independent of the potential impact of vaccinations.

### Odds reduction rates (ORR)

Simulating a clinical trials design, we chose risk reduction rate, commonly used to evaluate the treatment efficacy in clinical trials as the key measure. As this is a retrospective study, the term “odds reduction rate” (ORR) was adopted.

$$ \begin{gathered} ORR \, = \, \left( {Rate_{Control} {-} \, Rate_{Drug} } \right) /Rate_{Control} \hfill \\ ORR_{Adj} = \, \left( {Rate_{Control} {-} \, Rate_{Drug} } \right) /Rate_{Control} * \, Risk_{Adj} \hfill \\ Rate_{Control} : Control \, group \, COVID{ - }19 \, rate \hfill \\ Rate_{Drug} : Drug \, group \, COVID{ - }19 \, rate \hfill \\ Risk_{Adj} : Risk \, adjustment \, factor \hfill \\ \end{gathered} $$

### Risk adjustment

One common approach to reduce observational study biases between treatment and control due to confounding variables, such as demographics and other non-controllable factors, is propensity score matching^23^. Unfortunately, this may also increase model imbalance, inefficiency, dependence and bias^24^.

We designed a new bias reducing approach, a health risk adjustment using Optum ERG® (Episode Risk Groups) Risk Scores^10^. ERG uses a patient’s episodes of care, built by collecting all inpatient, outpatient, and ancillary services into mutually exclusive and exhaustive categories, focusing on the key information describing a patient’s underlying medical conditions including patient’s comorbidities and condition-specific complications, medications such as patient’s use of prescription drugs, and demographics including age and gender. Therefore, Risk adjustment using ERG Risk Scores may be able to reflect all available key clinical information to achieve more accurate prediction^10^.

We discovered that COVID-19 consequence rates are highly correlated (positively proportional) to Optum ERG® Retrospective Risks in a near perfect simple linear regression pattern across all study groups (Fig. 1), which allows Risk_Ratio for COVID-19 outcomes to be calculated directly from the regression slopes of corresponding COVID-19 Rate to Health Risk Figures (Figure S1-S5, Supplementary Information). Also, the more severe the COVID-19 outcomes, the steeper the regression slopes, the higher the Risk_Ratios (Death 1.04, ICU 0.83, Hospitalization 0.72, Pneumonia 0.48, Infection 0.17), which suggests that the higher the health risk, the higher risks/odds of more severe COVID-19 outcome.

$$RiskAdj = 1 + (RiskControl / RiskDrug - 1) * Risk\_Ratio$$

$$Risk\_Ratio = \%COVID\_Rate\_Change / \%Health\_Risk\_Change$$

$$ \begin{gathered} {\boldsymbol{y}} \, = \, {\boldsymbol{b}} \, * \, {\boldsymbol{X}} \, + {\boldsymbol{ e}} \hfill \\ y: \, COVID\_Rate \hfill \\ X: \, Health\_Risk \hfill \\ \% Rate\_Change = \left( {y2 \, - \, y1} \right) /y1 \left( {1: \, control, \, 2: \, drug/vaccine} \right) \hfill \\ \% Risk\_Change = \left( {X2 \, - \, X1} \right) /X1 \left( {1: \, control, \, 2: \, drug/vaccine} \right) \hfill \\ \end{gathered} $$

1 $$ \begin{gathered} Risk\_Ratio = \% Rate\_Change/\% Risk\_Change \hfill \\ = \left[ {\left( {y2 \, - \, y1} \right) /y1} \right]/ \, \left[ {\left( {X2 \, - \, X1} \right) /X1} \right] \hfill \\ = \, \left( {y2 \, - \, y1} \right) /\left( {X2 \, - \, X1} \right) \, * \, X1 /y1 \hfill \\ \end{gathered} $$

2 $$y1 = b * X1 + e$$

3 $$y2 = b * X2 + e$$

Equation (3) −Eq. (2)

$$ \begin{gathered} = = > \hfill \\ y2 \, - \, y1 \, = \, b \, * \, \left( {X2 \, - \, X1} \right) \hfill \\ = = > \hfill \\ \left( {y2 \, - \, y1} \right) /\left( {X2 \, - \, X1} \right) \, = \, b \hfill \\ = = > \hfill \\ {\text{Equation }}\left( 1 \right) \, = \, b \, * \, X1 /y1 \hfill \\ = = > \hfill \\ Risk\_Ratio = b \, * \, X1 /y1 \hfill \\ X1 \, = \, 1.79 \, \left( {control \, Risk,{{ {\rm Table\, S1}\!-\!{\rm cell\, I2}\!-\!{\rm I7}}}} \right) \hfill \\ \end{gathered} $$

- * Infection: b* = *0.00645 (Infection, *Fig. S5*), y1* = *0.0671 (control Infection, *Table S1*—cell K3)*
  $$ Risk\_Ratio\, = \,b \, * \, X1/y1\, = \,0.00645 \, * \, 1.79/0.0671\, = \,0.17. $$
- * Pneumonia: b* = *0.00410 (Pneumonia, *Fig. S3*), y1* = *0.0153 (control Pneumonia, *Table S1*—cell K7)*
  $$ Risk\_Ratio\, = \,b \, * \, X1/y1\, = \,0.00410 \, * \, 1.79/0.0153\, = \,0.48. $$
- *Hospitalization: b* = *0.00879 (Hospital, *Fig. S4*), y1* = *0.0219 (control Hospital, *Table S1*—cell K5)*
  $$ Risk\_Ratio\, = \,b \, * \, X1/y1\, = \,0.00879 \, * \, 1.79/0.0219\, = \,0.72. $$
- * ICU: b* = *0.00529 (ICU, *Fig. S2*), y1* = *0.0115 (control ICU, *Table S1*—cell K6)*
  $$ Risk\_Ratio\, = \,b \, * \, X1/y1\, = \,0.00529 \, * \, 1.79/0.0115\, = \,0.83. $$
- * Death: b* = *0.00223 (Death, *Fig. S1*), y1* = *0.00386 (control Death, *Table S1*—cell K4)*
  $$ Risk\_Ratio\, = \,b \, * \, X1/y1\, = \,0.00223 \, * \, 1.79/0.00386\, = \,1.04. $$

### Statistical significance

The statistical significance of odds reduction rates (ORR) on the COVID-19 outcome was calculated using AB Testing^25^. To counteract potential multiplicity errors caused by the multiple comparisons (multiple clinical trial end points—five COVID-19 consequences), Bonferroni Correction was applied: increase confidence interval (CI) from 95 to 99% (p-value decreased from 0.05 to 0.01). A one-sided hypothesis for statistical analysis was selected in this study driven by our specific interest and focus on positive outcomes that are superior to those observed in the control group, signifying favorable drug repurposing potential. Therefore, statistical significance was only calculated for the positive outcomes.

### Advantage of the study design

- Repurposing drugs with broad range of anti-COVID-19 mechanisms to help fight not only current COVID-19 variants but also future ones
- Large scale diverse cohorts quickly built on RWD
- Unique, straightforward and more accurate risk adjustment using Optum ERG Risk Scores to make all treatments and control comparable with one another
- Quickly and quantitatively identify potential drug/drug combination benefits
- Quickly generate RWE for drug repurposing to help design more focused clinical trials

### Limitation of the study design

- Observational study may not make definitive statements of fact about the drug efficacy and still needs to be validated via randomized controlled clinical trials though observational study could produce very similar results to randomized controlled clinical trials^20^.
- This study design did not exclude polypharmacy as almost all patients took more than only one drug and excluding polypharmacy will exclude almost all patients from drug groups.
- Due to the study window up to 2021-09-30, drug effects on Omicron *and later* variants cannot be evaluated. Further, this study did not distinguish variants in the analysis.
- Drug dosage effect was not studied in the analysis.

### Guideline/accordance statement

This is to confirm that all methods above were performed in accordance with the relevant guidelines and regulations as it’s a retrospective observational study on de-identified medical claims data and does not carry out experiments on humans and/or use human tissue samples. This de-identified data analytic study has been approved by Dr. Ashley Brenton, VP of Clinicogenomics, Life Sciences, OptumInsight/OptumRx, UnitedHealth Group.

### Supplementary Information

Supplementary Legends.

Supplementary Figure S1.

Supplementary Figure S2.

Supplementary Figure S3.

Supplementary Figure S4.

Supplementary Figure S5.

Supplementary Information.

Supplementary Table S1.

## Data Availability

The datasets generated and analysed during the current study are not publicly available due to the proprietary nature of the Optum datasets but can be accessible via requesting a license from Optum, UnitedHealth Group.

## Code availability

Source data used for this study are from Optum Clinformatics™ Data Mart^26^. Source codes are provided in a separated file—“General sample codes”.
