# Supplementary material for: Identification of potential new COVID-19 treatments via RWD-driven drug repurposing
Source: Sci Rep. 2023 Sep 4;13:14586. doi: 10.1038/s41598-023-40033-8 (PMC10477169; doi:10.1038/s41598-023-40033-8)
Supplement: Supplementary file 1 — Supplementary Legends. [file 41598_2023_40033_MOESM1_ESM.docx]

**Table S1** **|** **Top Repurposing Drugs - Result Details**

Statistical significance was calculated only for positive outcomes (one-sided), of study interest and focus.

**Figure S1 | Line Fit Plot - COVID Death Rate (y) vs Health Risk (X): y = b*X + e**

R Square: 0.993306122; P-value: 1.68406E-05;

Coefficients: Intercept (e) = -0.0002, Regression Slope (b) = 0.00222857

**Figure S2 | Line Fit Plot - COVID ICU Rate (y) vs Health Risk (X): y = b*X + e**

R Square: 0.995387955; P-value: 7.9889E-06;

Coefficients: Intercept (e) = 0.001586667, Regression Slope (b) = 0.005294286

**Figure S3 | Line Fit Plot - COVID Pneumonia Rate (y) vs Health Risk (X): y = b*X + e**

R Square: 0.997640232; P-value: 2.08983E-06;

Coefficients: Intercept (e) = 0.00896, Regression Slope (b) = 0.004097143

**Figure S4 | Line Fit Plot - COVID Hospitalization Rate (y) vs Health Risk (X): y = b*X + e**

R Square: 0.997860802; P-value: 1.71729E-06;

Coefficients: Intercept (e) = 0.00784, Regression Slope (b) = 0.008788571

**Figure S5 | Line Fit Plot - COVID Infection Rate (y) vs Health Risk (X): y = b*X + e**

R Square: 0.996202966; P-value: 5.41341E-06;

Coefficients: Intercept (e) = 0.05878, Regression Slope (b) = 0.006448571
