# Supplementary figures and images for: Identification of potential new COVID-19 treatments via RWD-driven drug repurposing
Source: Sci Rep. 2023 Sep 4;13:14586. doi: 10.1038/s41598-023-40033-8 (PMC10477169; doi:10.1038/s41598-023-40033-8)

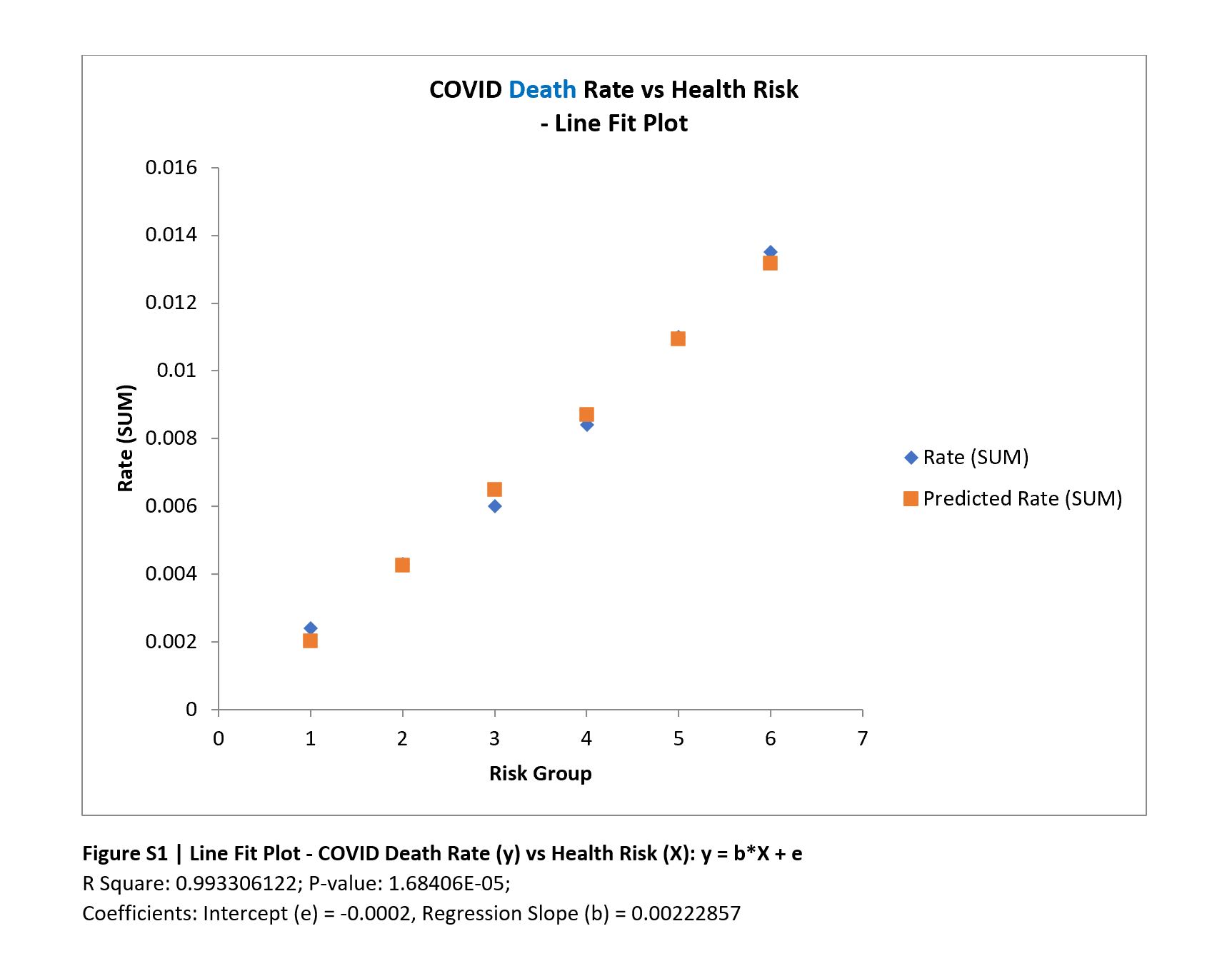

Supplement: Supplementary file 2 — Supplementary Figure S1. [file 41598_2023_40033_MOESM2_ESM.jpg]

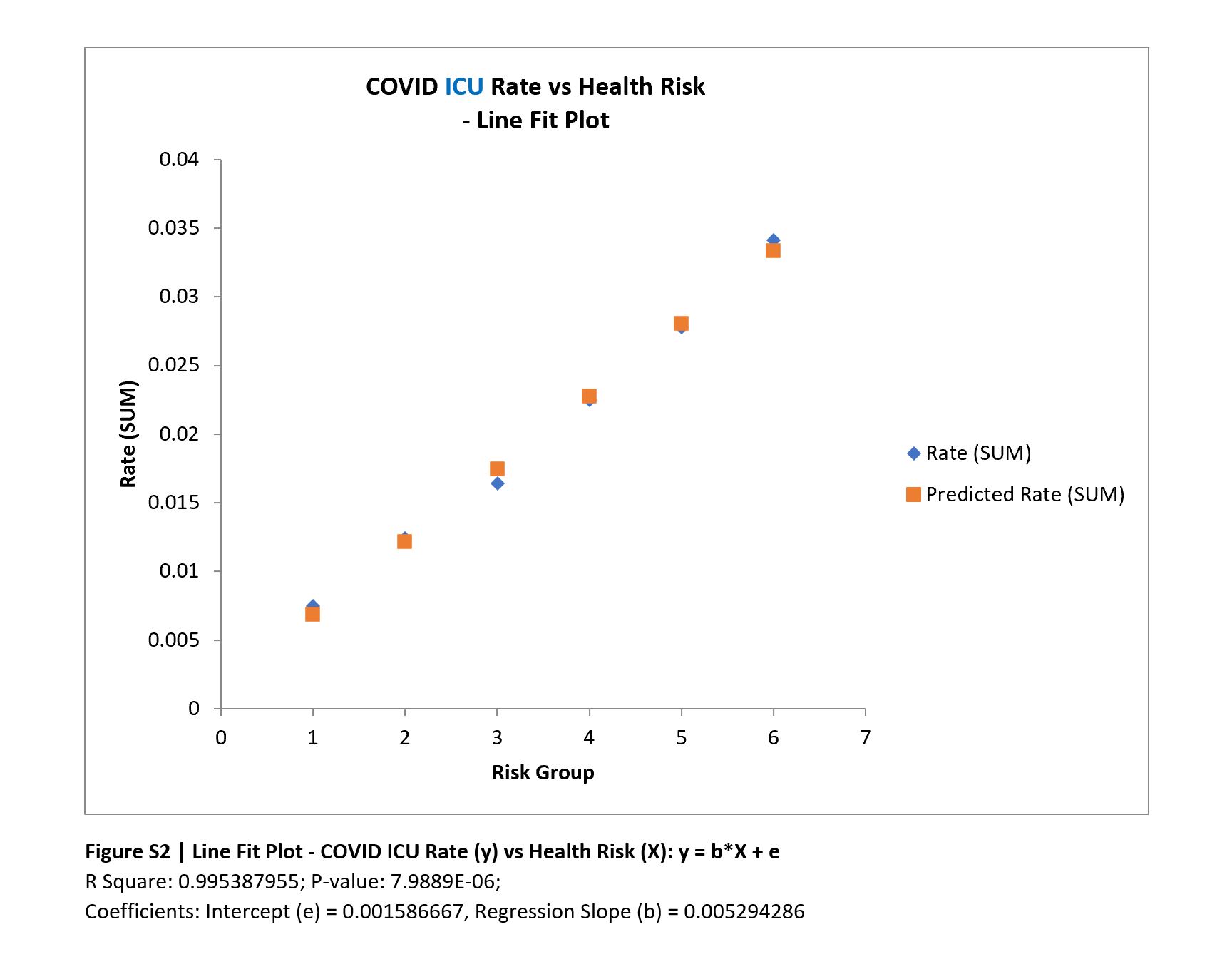

Supplement: Supplementary file 3 — Supplementary Figure S2. [file 41598_2023_40033_MOESM3_ESM.jpg]

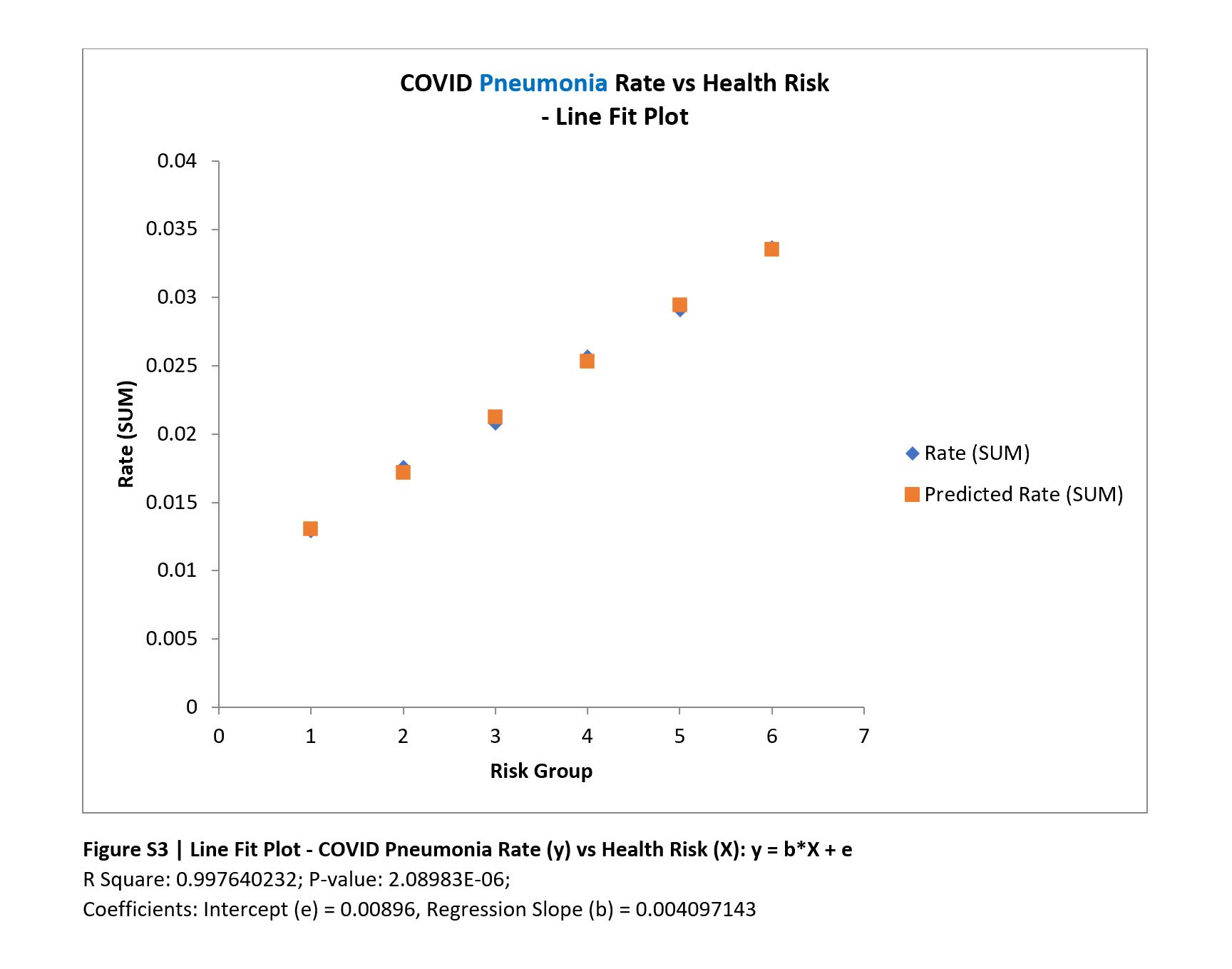

Supplement: Supplementary file 4 — Supplementary Figure S3. [file 41598_2023_40033_MOESM4_ESM.jpg]

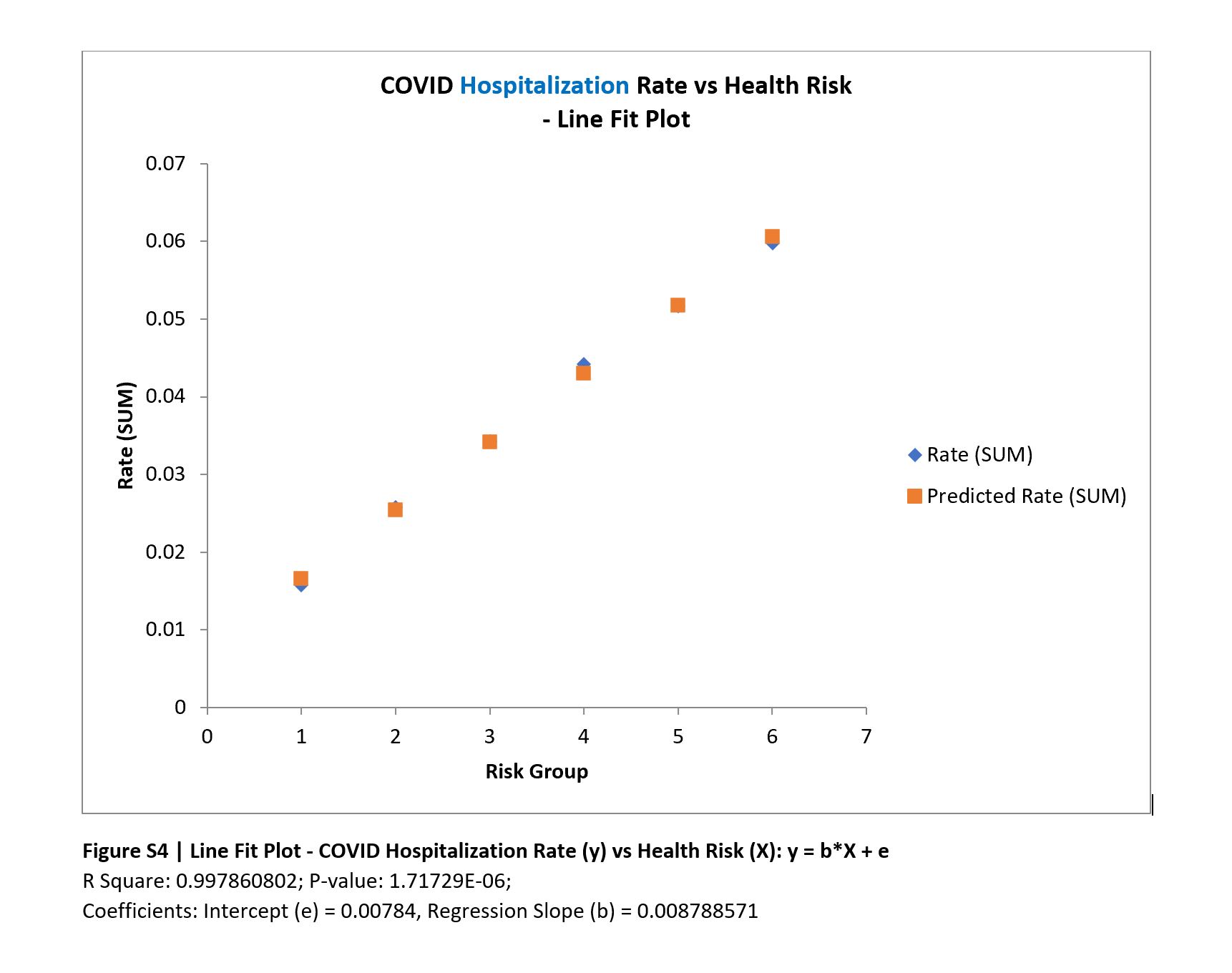

Supplement: Supplementary file 5 — Supplementary Figure S4. [file 41598_2023_40033_MOESM5_ESM.jpg]

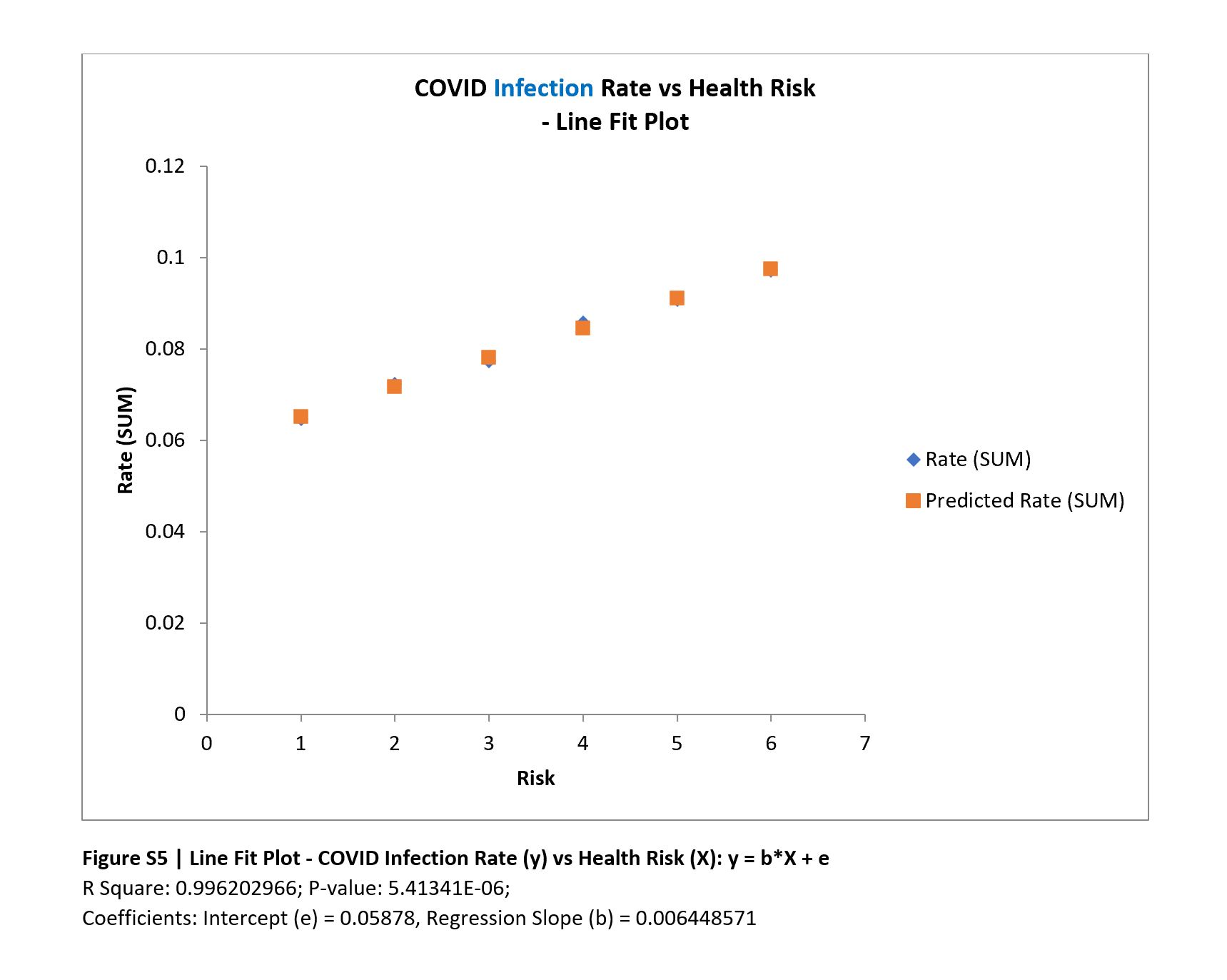

Supplement: Supplementary file 6 — Supplementary Figure S5. [file 41598_2023_40033_MOESM6_ESM.jpg]
