# Supplementary material for: Identification of potential new COVID-19 treatments via RWD-driven drug repurposing
Source: Sci Rep. 2023 Sep 4;13:14586. doi: 10.1038/s41598-023-40033-8 (PMC10477169; doi:10.1038/s41598-023-40033-8)
Supplement: Supplementary file 7 — Supplementary Information. [file 41598_2023_40033_MOESM7_ESM.docx]

General sample codes

-- Control group - All patients Non-vaccinated with Covid exposure before Covid infection plus non-infected

DROP TABLE NonVaccination1;

CREATE TABLE NonVaccination1 AS

SELECT distinct a.patient_id FROM member_table a

join pharmacy_table b on a.patient_id = b.patient_id

join diagnosis_table c on c.patient_id = b.patient_id

where ndc not in (COVID vaccine NDCs)

and icd_flag = '10' AND diag in ('Z20822')

and (exists (

select patient_id from diagnosis_table d

where d.icd_flag = '10' AND d.diag in ('U071')

and d.patient_id=c.patient_id

and d.fst_dt > c.fst_dt)

or not exists (

select patient_id from diagnosis_table d

where d.icd_flag = '10' AND d.diag in ('U071')

and d.patient_id=c.patient_id));

SELECT count (distinct patient_id) FROM NonVaccination1; -- 2,935,415

DROP TABLE NonVaccination1_R;

CREATE TABLE NonVaccination1_R AS

SELECT 'Covid-19 Infection' as Category, count(distinct a.patient_id) as Patients, count(distinct a.patient_id)/2935415e0*100 as Percentage

FROM "diagnosis" a join NonVaccination1 c on a.patient_id = c.patient_id

WHERE icd_flag = '10' AND diag in ('U071')

union

SELECT 'COVID-19 exposure/contact' as Category, count(distinct a.patient_id) as Patients,count(distinct a.patient_id)/2935415e0*100 as Percentage

FROM "diagnosis" a join NonVaccination1 c on a.patient_id = c.patient_id

WHERE icd_flag = '10' AND diag in ('Z20822')

union

SELECT 'Pneumonia due to COVID-19' as Category, count(distinct a.patient_id) as Patients, count(distinct a.patient_id)/2935415e0*100 as Percentage

FROM "diagnosis" a join NonVaccination1 c on a.patient_id = c.patient_id

WHERE icd_flag = '10' AND diag in ('J1282')

union

select 'Hospitalized' as Category, count(distinct b.patient_id) as Patients,count(distinct b.patient_id)/2935415e0*100 as Percentage

FROM "confinement" a join "diagnosis" b

on a.patient_id = b.patient_id join NonVaccination1 c on a.patient_id = c.patient_id

WHERE a.admit_date >= c.diag_dt - interval '14' day and b.icd_flag = '10' AND diag in ('U071')

Union -- 14-day incubation period added in case of delayed diag of COVID after admission

select 'ICU' as Category, count(distinct b.patient_id) as Patients,count(distinct b.patient_id)/2935415e0*100 as Percentage

FROM "confinement" a join "diagnosis" b

on a.patient_id = b.patient_id join NonVaccination1 c on a.patient_id = c.patient_id

WHERE a.admit_date >= c.diag_dt - interval '14' day and a.ICU_ind = 'Y' and b.icd_flag = '10' AND diag in ('U071') -- 14-day incubation period added in case of delayed diag of COVID after admission

union

select 'Deceased' as Category, count(distinct b.patient_id) as Patients,count(distinct b.patient_id)/2935415e0*100 as Percentage

FROM "death_2007" a join "diagnosis" b

on a.patient_id = b.patient_id join NonVaccination1 c on a.patient_id = c.patient_id

WHERE b.icd_flag = '10' AND diag in ('U071') -- and date(date_parse(ymdod, '%Y%m')) >= date('2020-01-01')

;

select * from NonVaccination1_R;

category patients percentage

Deceased 11319 0.38560135449331695

ICU 33679 1.1473335116159045

Pneumonia due to COVID-19 45055 1.5348766699086842

Hospitalized 64395 2.1937272923930684

Covid-19 Infection 196847 6.705934254611358

COVID-19 exposure/contact 2935415 100.0

-- Reference group - Vaccinated at least 7 weeks before Diag of Covid exposure and infection occurred after or no infection

DROP TABLE Vaccination1;

CREATE TABLE Vaccination1 AS

SELECT distinct a.patient_id, 2021 - d."yrdob" as age,

case d."race" when 'A' then 2 when 'B' then 4 when 'W' then 1 else 3 end as race,

case d."gdr_cd" when 'M' then 1 when 'F' then 0 else 0.5 end as gender

FROM "pharmacy" a

join "diagnosis" b

on a.patient_id = b.patient_id

left join "mbr_enroll_2007" d

on a.patient_id = d.patient_id

where ndc in ('59676058015', '59676058005', '80777027399', '80777027398', '80777027310',

'80777027315', '59267100002', '59267100003', '59267100001', '59267102502', '59267102503',

'59267102504', '59267102501','59267105502', '59267105504', '59267105501','59267007802',

'59267007804', '59267007801','00069100002', '00069100003', '00069100001')

and fill_dt <= (fst_dt-interval '50' day)

and icd_flag = '10' AND diag in ('Z20822')

and (exists (

select patient_id from "diagnosis" d

where d.icd_flag = '10' AND d.diag in ('U071')

and d.patient_id=b.patient_id

and d.fst_dt > b.fst_dt) -- 1) make sure covid infection happened after covid exposure

or not exists ( -- 2) include non-infection after covid exposure

select patient_id from "diagnosis" d

where d.icd_flag = '10' AND d.diag in ('U071')

and d.patient_id=b.patient_id))

;

select count (distinct patient_id) from Vaccination1; -- 189,692

-- 1st shot to 2nd shot: 3 weeks

-- Add COVID Exposure/Infection date

DROP TABLE Vaccination1_1;

CREATE TABLE Vaccination1_1 AS

SELECT a.*, case when c.diag in ('Z20822','U071') then c.fst_dt end as diag_dt

FROM Vaccination1 a

left join "diagnosis" c

on c.patient_id = a.patient_id

and icd_flag = '10' AND diag in ('Z20822','U071') -- covid exposure/infection

group by 1,2,3,4,5;

select count (distinct patient_id) from Vaccination1; -- 189,692

DROP TABLE Vaccination1;

CREATE TABLE Vaccination1 AS

select * from Vaccination1_1

select count (distinct patient_id) from Vaccination1; -- 189,692

DROP TABLE Vaccination1_R;

CREATE TABLE Vaccination1_R AS --RiskAdj = 1 + (RiskControl / RiskDrug - 1) * Risk_Ratio

SELECT 'Covid-19 Infection' as Category, ceiling(count(distinct b.patient_id)*(1+(1.7911/1.4935-1)*0.17)) as Patients,

count(distinct b.patient_id)/189692e0*100*(1+(1.7911/1.4935-1)*0.17) as Percentage

FROM "diagnosis" a join Vaccination1 b

on a.patient_id = b.patient_id

WHERE icd_flag = '10' AND diag in ('U071')

union

SELECT 'COVID-19 exposure/contact' as Category, count(distinct b.patient_id) Patients,

count(distinct b.patient_id)/189692e0*100 as Percentage

FROM "diagnosis" a join Vaccination1 b

on a.patient_id = b.patient_id

WHERE icd_flag = '10' AND diag in ('Z20822')

union

SELECT 'Pneumonia due to COVID-19' as Category, ceiling(count(distinct b.patient_id)*(1+(1.7911/1.4935-1)*0.48)) as Patients,

count(distinct b.patient_id)/189692e0*100*(1+(1.7911/1.4935-1)*0.48) as Percentage

FROM "diagnosis" a join Vaccination1 b

on a.patient_id = b.patient_id

WHERE icd_flag = '10' AND diag in ('J1282')

union

select 'Hospitalized' as Category, ceiling(count(distinct b.patient_id)*(1+(1.7911/1.4935-1)*0.72)) as Patients,

count(distinct b.patient_id)/189692e0*100*(1+(1.7911/1.4935-1)*0.72) as Percentage

FROM "confinement" a join Vaccination1 b

on a.patient_id = b.patient_id join "diagnosis" c on c.patient_id = b.patient_id

WHERE a.admit_date >= b.diag_dt - interval '14' day and c.icd_flag = '10' AND diag in ('U071')

union

select 'ICU' as Category, ceiling(count(distinct b.patient_id)*(1+(1.7911/1.4935-1)*0.83)) as Patients,

count(distinct b.patient_id)/189692e0*100*(1+(1.7911/1.4935-1)*0.83) as Percentage

FROM "confinement" a join Vaccination1 b

on a.patient_id = b.patient_id join "diagnosis" c on c.patient_id = b.patient_id

WHERE a.admit_date >= b.diag_dt - interval '14' day and ICU_ind = 'Y' and c.icd_flag = '10' AND diag in ('U071')

union

select 'Deceased' as Category, ceiling(count(distinct b.patient_id)*(1+(1.7911/1.4935-1)*1.04)) as Patients,

count(distinct b.patient_id)/189692e0*100*(1+(1.7911/1.4935-1)*1.04) as Percentage

FROM "death_2007" a join Vaccination1 b

on a.patient_id = b.patient_id join "diagnosis" c on c.patient_id = b.patient_id

WHERE c.icd_flag = '10' AND diag in ('U071') -- and date(date_parse(ymdod, '%Y%m')) >= date('2020-01-01')

;

select * from Vaccination1_R;

category patients percentage

Deceased 9.0 0.004454925931734873

ICU 217.0 0.11427065731793891

Pneumonia due to COVID-19 265.0 0.1391997547575951

Hospitalized 483.0 0.25438300732861746

Covid-19 Infection 4497.0 2.3703274070678075

COVID-19 exposure/contact 189692.0 100.0

-- Alinia

DROP TABLE Nitazoxanide;

CREATE TABLE Nitazoxanide AS

SELECT distinct a.patient_id , 2021 - d."yrdob" as age,

case d."race" when 'A' then 2 when 'B' then 4 when 'W' then 1 else 3 end as race,

case d."gdr_cd" when 'M' then 1 when 'F' then 0 else 0.5 end as gender

FROM "pharmacy" a

join "diagnosis" b on a.patient_id = b.patient_id

join NonVaccination1 c on b.patient_id = c.patient_id

left join "mbr_enroll_2007" d

on a.patient_id = d.patient_id

where (upper(gnrc_nm) like '%NITAZOXANIDE%'

or upper(brnd_nm) like '%NITAZOXANIDE%'

or upper(gnrc_nm) like '%ALINIA%'

or upper(brnd_nm) like '%ALINIA%'

or upper(gnrc_nm) like '%NIZONIDE%'

or upper(brnd_nm) like '%NIZONIDE%'

--or upper(gnrc_nm) in ('DXM')

--or upper(brnd_nm) in ('DXM')

)

and fill_dt >= date('2019-12-25')

and fill_dt between (fst_dt-interval '14' day) and (fst_dt+interval '50' day)

and icd_flag = '10' AND diag in ('Z20822')

and (exists (

select patient_id from "diagnosis" d

where d.icd_flag = '10' AND d.diag in ('U071')

and d.patient_id=b.patient_id

and d.fst_dt > b.fst_dt)

or not exists (

select patient_id from "diagnosis" d

where d.icd_flag = '10' AND d.diag in ('U071')

and d.patient_id=b.patient_id));

select count (distinct patient_id) from Nitazoxanide; -- 167

-- Add COVID Exposure/Infection date

DROP TABLE Nitazoxanide_1;

CREATE TABLE Nitazoxanide_1 AS

SELECT a.*, case when c.diag in ('Z20822','U071') then c.fst_dt end as diag_dt

FROM Nitazoxanide a

left join "diagnosis" c

on c.patient_id = a.patient_id

and icd_flag = '10' AND diag in ('Z20822','U071') -- covid exposure/infection

group by 1,2,3,4,5;

select count (distinct patient_id) from Nitazoxanide; -- 167

DROP TABLE Nitazoxanide;

CREATE TABLE Nitazoxanide AS

select * from Nitazoxanide_1

select count (distinct patient_id) from Nitazoxanide; -- 167

DROP TABLE Nitazoxanide_R;

CREATE TABLE Nitazoxanide_R AS --Co-Eff_drug = 1 + (Risk_nonVac / Risk_drug - 1)* AvgRate

SELECT 'Covid-19 Infection' as Category, ceiling(count(distinct b.patient_id)*(1+(1.7911/4.091463414634147-1)*0.17)) as Patients,

count(distinct b.patient_id)/167e0*100*(1+(1.7911/4.091463414634147-1)*0.17) as Percentage

FROM "diagnosis" a join Nitazoxanide b

on a.patient_id = b.patient_id

WHERE icd_flag = '10' AND diag in ('U071')

union

SELECT 'COVID-19 exposure/contact' as Category, count(distinct b.patient_id) Patients,

count(distinct b.patient_id)/167e0*100 as Percentage

FROM "diagnosis" a join Nitazoxanide b

on a.patient_id = b.patient_id

WHERE icd_flag = '10' AND diag in ('Z20822')

union

SELECT 'Pneumonia due to COVID-19' as Category, ceiling(count(distinct b.patient_id)*(1+(1.7911/4.091463414634147-1)*0.48)) as Patients,

count(distinct b.patient_id)/167e0*100*(1+(1.7911/4.091463414634147-1)*0.48) as Percentage

FROM "diagnosis" a join Nitazoxanide b

on a.patient_id = b.patient_id

WHERE icd_flag = '10' AND diag in ('J1282')

union

select 'Hospitalized' as Category, ceiling(count(distinct b.patient_id)*(1+(1.7911/4.091463414634147-1)*0.72)) as Patients,

count(distinct b.patient_id)/167e0*100*(1+(1.7911/4.091463414634147-1)*0.72) as Percentage

FROM "confinement" a join Nitazoxanide b

on a.patient_id = b.patient_id join "diagnosis" c on c.patient_id = b.patient_id

WHERE a.admit_date >= b.diag_dt - interval '14' day and c.icd_flag = '10' AND diag in ('U071')

union

select 'ICU' as Category, ceiling(count(distinct b.patient_id)*(1+(1.7911/4.091463414634147-1)*0.83)) as Patients,

count(distinct b.patient_id)/167e0*100*(1+(1.7911/4.091463414634147-1)*0.83) as Percentage

FROM "confinement" a join Nitazoxanide b

on a.patient_id = b.patient_id join "diagnosis" c on c.patient_id = b.patient_id

WHERE a.admit_date >= b.diag_dt - interval '14' day and ICU_ind = 'Y' and c.icd_flag = '10' AND diag in ('U071')

union

select 'Deceased' as Category, ceiling(count(distinct b.patient_id)*(1+(1.7911/4.091463414634147-1)*0.94)) as Patients,

count(distinct b.patient_id)/167e0*100*(1+(1.7911/4.091463414634147-1)*0.94) as Percentage

FROM "death_2007" a join Nitazoxanide b

on a.patient_id = b.patient_id join "diagnosis" c on c.patient_id = b.patient_id

WHERE c.icd_flag = '10' AND diag in ('U071') -- and date(date_parse(ymdod, '%Y%m')) >= date('2020-01-01')

;

select * from Nitazoxanide_R;

category patients percentage

Deceased 0.0 0.0

ICU 2.0 0.6387365929839278

Pneumonia due to COVID-19 2.0 0.874403905155412

Hospitalized 3.0 1.4256069250470744

Covid-19 Infection 14.0 8.123533576661877

COVID-19 exposure/contact 167.0 100.0

-- ERG Risk by Drug

DROP TABLE ERG_Risk;

CREATE TABLE ERG_Risk AS

select '*Control: Non-Vaccination' as Drug, avg(risk) as Risk_Score from

(select distinct b.patient_id, avg(retrospective_risk) risk

from NonVaccination1 a

join "erg_risk_record" b

on a.patient_id=b.patient_id group by b.patient_id

)

--1.7911537655490386

union

select '*Ref: COVID-19 Vaccines' as Drug, avg(risk) as Risk_Score from

(select distinct b.patient_id, avg(retrospective_risk) risk

from Vaccination1 a

join "erg_risk_record" b

on a.patient_id=b.patient_id group by b.patient_id

)

--1.4935191049187249

union

select 'Alinia' as Drug, avg(risk) as Risk_Score from

(select distinct b.patient_id, avg(retrospective_risk) risk

from Nitazoxanide a

join "erg_risk_record" b

on a.patient_id=b.patient_id group by b.patient_id

)

--4.091463414634147

--ERG Risk vs COVID Outcome

DROP TABLE risk_outcome;

CREATE TABLE risk_outcome AS

with tab1 as --nonVac risk group

(

select count(distinct b.patient_id) patients,

case retrospective_risk when 0 then '0' when 1 then '01' when 2 then '02' when 3 then '03' when 4 then '04'

when 5 then '05' when 6 then '06' when 7 then '07' when 8 then '08' when 9 then '09' when 10 then '10'

when 11 then '11' when 12 then '12' when 13 then '13' when 14 then '14' when 15 then '15' when 16 then '16'

else '>16' end as risk_group

from NonVaccination1 a

join "erg_risk_record" b on a.patient_id=b.patient_id

group by 2),

tab2 as -- deseased risk group

(

select count(distinct b.patient_id) patients,

case retrospective_risk when 0 then '0' when 1 then '01' when 2 then '02' when 3 then '03' when 4 then '04'

when 5 then '05' when 6 then '06' when 7 then '07' when 8 then '08' when 9 then '09' when 10 then '10'

when 11 then '11' when 12 then '12' when 13 then '13' when 14 then '14' when 15 then '15' when 16 then '16'

else '>16' end as risk_group

from NonVaccination1 a

join "erg_risk_record" b

on a.patient_id=b.patient_id

join "diagnosis" c

on c.patient_id = b.patient_id

join "death_2007" d

on c.patient_id = d.patient_id

WHERE c.icd_flag = '10' AND c.diag in ('U071')

group by 2),

tab3 as -- ICU risk group

(

select count(distinct b.patient_id) patients,

case retrospective_risk when 0 then '0' when 1 then '01' when 2 then '02' when 3 then '03' when 4 then '04'

when 5 then '05' when 6 then '06' when 7 then '07' when 8 then '08' when 9 then '09' when 10 then '10'

when 11 then '11' when 12 then '12' when 13 then '13' when 14 then '14' when 15 then '15' when 16 then '16'

else '>16' end as risk_group

from NonVaccination1 a

join "erg_risk_record" b

on a.patient_id=b.patient_id

join "diagnosis" c

on c.patient_id = b.patient_id

join "confinement" d

on c.patient_id = d.patient_id

WHERE d.admit_date >= a.diag_dt - interval '14' day and d.ICU_ind = 'Y' and c.icd_flag = '10' AND c.diag in ('U071')

group by 2),

tab4 as -- Hospitalized risk group

(

select count(distinct b.patient_id) patients,

case retrospective_risk when 0 then '0' when 1 then '01' when 2 then '02' when 3 then '03' when 4 then '04'

when 5 then '05' when 6 then '06' when 7 then '07' when 8 then '08' when 9 then '09' when 10 then '10'

when 11 then '11' when 12 then '12' when 13 then '13' when 14 then '14' when 15 then '15' when 16 then '16'

else '>16' end as risk_group

from NonVaccination1 a

join "erg_risk_record" b

on a.patient_id=b.patient_id

join "diagnosis" c

on c.patient_id = b.patient_id

join "confinement" d

on c.patient_id = d.patient_id

WHERE d.admit_date >= a.diag_dt - interval '14' day and c.icd_flag = '10' AND c.diag in ('U071')

group by 2),

tab5 as -- Pneumonia risk group

(

select count(distinct b.patient_id) patients,

case retrospective_risk when 0 then '0' when 1 then '01' when 2 then '02' when 3 then '03' when 4 then '04'

when 5 then '05' when 6 then '06' when 7 then '07' when 8 then '08' when 9 then '09' when 10 then '10'

when 11 then '11' when 12 then '12' when 13 then '13' when 14 then '14' when 15 then '15' when 16 then '16'

else '>16' end as risk_group

from NonVaccination1 a

join "erg_risk_record" b

on a.patient_id=b.patient_id

join "diagnosis" c

on c.patient_id = a.patient_id

WHERE c.icd_flag = '10' AND c.diag in ('J1282')

group by 2),

tab6 as -- Infection risk group

(

select count(distinct b.patient_id) patients,

case retrospective_risk when 0 then '0' when 1 then '01' when 2 then '02' when 3 then '03' when 4 then '04'

when 5 then '05' when 6 then '06' when 7 then '07' when 8 then '08' when 9 then '09' when 10 then '10'

when 11 then '11' when 12 then '12' when 13 then '13' when 14 then '14' when 15 then '15' when 16 then '16'

else '>16' end as risk_group

from NonVaccination1 a

join "erg_risk_record" b

on a.patient_id=b.patient_id

join "diagnosis" c

on c.patient_id = a.patient_id

WHERE c.icd_flag = '10' AND c.diag in ('U071')

group by 2)

select tab1.risk_group as risk_group, 0.1*10*tab2.patients/tab1.patients as outcome_rate, 'Deceased' as category

from tab1 join tab2 on tab1.risk_group = tab2.risk_group

union

select tab1.risk_group as risk_group, 0.1*10*tab3.patients/tab1.patients as outcome_rate, 'ICU' as category

from tab1 join tab3 on tab1.risk_group = tab3.risk_group

union

select tab1.risk_group as risk_group, 0.1*10*tab4.patients/tab1.patients as outcome_rate, 'Hospitalized' as category

from tab1 join tab4 on tab1.risk_group = tab4.risk_group

union

select tab1.risk_group as risk_group, 0.1*10*tab5.patients/tab1.patients as outcome_rate, 'Pneumonia due to COVID-19' as category

from tab1 join tab5 on tab1.risk_group = tab5.risk_group

union

select tab1.risk_group as risk_group, 0.1*10*tab6.patients/tab1.patients as outcome_rate, 'Covid-19 Infection' as category

from tab1 join tab6 on tab1.risk_group = tab6.risk_group;

select * from risk_outcome;
